# Study on Synergistic Effect of Xanthan Gum and Sodium Methylsiliconate on Mechanical Strength and Water Stability of Phosphogypsum Road-Based Materials

**DOI:** 10.3390/ma16206766

**Published:** 2023-10-19

**Authors:** Jianhui Wu, Tong Xu, Hongqiang Chu, Xiang Xi, Fengchen Zhang, Weizhun Jin

**Affiliations:** College of Mechanics and Materials, Hohai University, Nanjing 211100, China; 211308020017@hhu.edu.cn (J.W.); 221308030005@hhu.edu.cn (T.X.); chq782009@126.com (H.C.); xiangxi93@hhu.edu.cn (X.X.); j_weizhun@163.com (W.J.)

**Keywords:** phosphogypsum, solid waste recycling, xanthan gum, road-based material, hazardous substance

## Abstract

To address the issues of low strength, poor water stability, and hazardous substance leaching associated with using phosphogypsum (PG) as a direct road-based material, the traditional approach involves employing inorganic cementing materials to stabilize PG, effectively addressing the problems. This study innovatively utilizes the xanthan gum (XG) and sodium methylsiliconate (SM) as curing agents for PG to solve the above problems. An organic curing agent stabilized PG was prepared by dry mixing XG and PG. The unconfined compressive strength, water stability, and leaching behavior of stabilized PG were investigated, the leaching behavior was characterized by ion leaching concentration, and the mechanisms behind the strength development of stabilized PG were explored by SEM and FTIR. The experimental results indicate that the single incorporation of XG reduced the strength and water stability of stabilized PG, while the single incorporation of SM had a limited effect on strength and water stability. In addition, the dual incorporation of XG and SM significantly improved the strength and water stability of stabilized PG. At the same time, the dual incorporation of XG and SM greatly reduced the leaching of hazardous substances from stabilized PG. These results demonstrate the feasibility of using stabilized PG for road base materials.

## 1. Introduction

PG (a byproduct of the phosphate fertilizer industry) is an accumulation of solid waste, which is composed of calcium sulfate dihydrate (CaSO_4_·2H_2_O) with a weight content exceeding 90%. It suffers from impurities such as water-soluble phosphates, water-soluble fluorides, organic matter, and toxic metals [1,2], resulting in a low utilization rate, consequent land occupation, and a potential threat to atmospheric pollution due to the presence of fluoride, toxic metals, and radioactive particles. According to the statistics, about 80 million tons of PG are produced in China [3], for which the utilization rate is no more than 27% [4]. Moreover, the storage of the PG could cause the leakage of contaminants such as sulfates, phosphate ions, trace metals, and radioactive nuclides [5], posing a threat to groundwater. At present, the PG dominates in soil improvement [6,7,8,9,10], construction materials [1,11,12,13], and as a retarder in cement production [14,15,16,17,18].

Solid waste recycling is of utmost importance in modern environmental conservation efforts. Reusing solid waste can alleviate pressure on ponds and landfills and reduce the financial burden of environmental protection [19]. Recycling resources reduces energy consumption for raw construction materials and associated carbon dioxide in the environment [20]. Using solid waste (PG) as a component in roadbed materials is promising due to the potential consumption of substantial quantities of this waste, and thus the conservation of non-renewable sand and gravel resources [21,22,23,24,25]. However, the primary component in PG (CaSO_4_·2H_2_O) can dissolve in the presence of water. Consequently, the harmful substances would leach out [26], causing contamination to the soil and water systems [27,28]. As a result, it is necessary to deal with corresponding issues of direct utilization of PG in roadbed materials [29,30].

The utilization of PG in road construction has been subject to extensive investigation. A compacted model was developed by D. Moussa et al. [31] to study the mechanical behavior of PG. The results showed that the strength is attributed to the existing of inherent cohesion and additional cohesion caused by capillary effect when the water content is low. However, this additional cohesion vanishes when immersed in water, resulting in a decrease in strength. Meskini et al. [32] found that the incorporation of fly ash and lime can increase the unconfined compressive strength and enhance durability of the PG composite due to the formation of ettringite as the main hydration product responsible for strength development. Ngo et al. [33] demonstrated that the stabilization of a PG and fly ash mixture, along with curing using cement and lime, leads to improved mechanical properties of PG. This approach confirms the practical feasibility of employing PG as a filling material for road base construction. Shen et al. [34] prepared PG stabilized by steel slag and fly ash to be utilized as road base material, showing that the early strength of which is higher than lime-fly ash and lime-soil road base materials, and the long-term strength is much higher than cement stabilized granular materials.

Currently, the research on PG road base materials predominantly focuses on the use of traditional inorganic stabilizers, such as lime and cement, to strengthen and stabilize PG. However, the utilization of these traditional stabilizers gives rise to various environmental concerns, including CO_2_ emissions from cement production, hinderance of vegetation growth, groundwater contamination, and the creation of heat islands [35]. The organic stabilizer is a promising alternative in this regard. However, the research of organic stabilizers is limited. XG has been extensively studied as a stabilizing agent for soil, and previous research has shown that microorganisms produce xanthan gum, which aids in soil aggregation, biologging, and bio-cementation, and can be beneficial for stabilization, liquefaction mitigation, strengthening tailing dams, and binding, among other applications [35,36,37,38,39]. Despite its proven success as a soil stabilizer, the potential application of xanthan gum for stabilizing PG remains unexplored.

As previously highlighted, the direct application of PG or its stabilization using conventional inorganic materials for roadbed construction presents certain limitations. To address these shortcomings, this study introduces an innovative approach, employing XG and SM as organic curing agents to stabilize PG for roadbed applications. The experimental procedure commenced with an assessment of the composition, mineral structure, and micro-morphology of PG. Subsequently, the study shifted its focus to investigate the unconfined compressive strength of stabilized PG. This encompassed an examination of not only the impact of curing agent content on unconfined compressive strength but also a consideration of the effects of curing time and temperature on unconfined compressive strength, along with a thorough exploration of the mechanisms underlying strength development. Following the unconfined compressive strength analysis, the research extended to investigate the effects of dosage, curing time, and temperature on water stability. Finally, the environmental impact of stabilized PG was assessed through the analysis of the leaching concentration of ions and elements. The culmination of these findings substantiates the feasibility of utilizing stabilized PG as a material for pavement base construction.

## 2. Materials and Experimental Procedure

### 2.1. Materials

#### 2.1.1. PG

**Figure 1:**
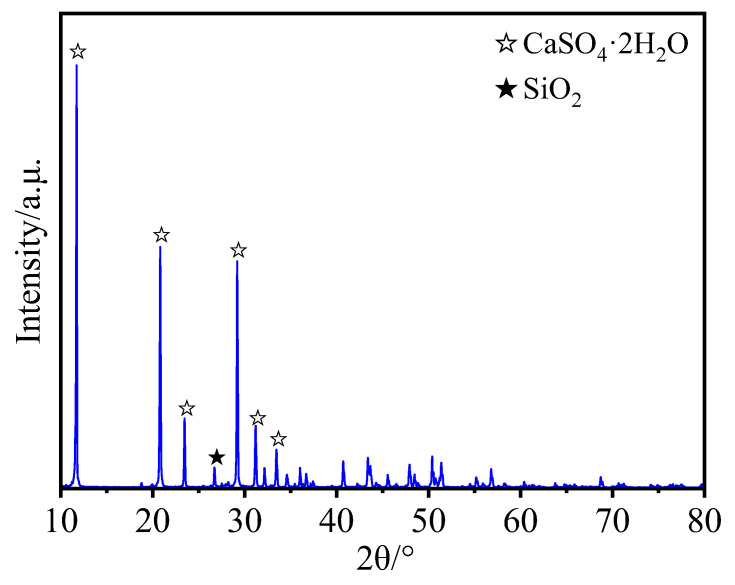
X-ray diffraction of PG for 2θ.

**Figure 2:**
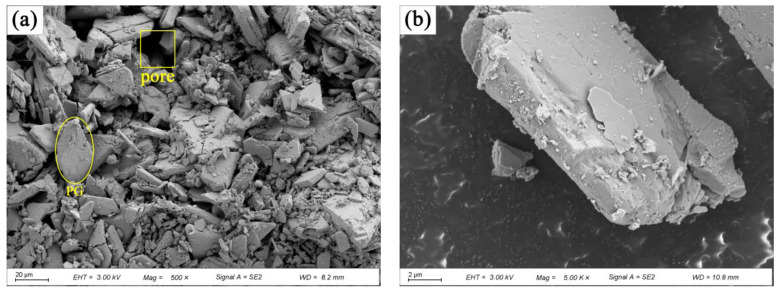
Morphology of PG: (**a**) 500× (20 μm); (**b**) 5000× (2 μm).

PG was kindly provided by Anhui Liuguo Chemical Co., Ltd. (Tongling, China). It exhibits weak acidic characteristics, as indicated by the manufacturer. The chemical composition of PG, determined via X-ray fluorescence spectroscopy (XRF) analysis and Ion Chromatography (IC), is presented in Table 1. The major constituents of PG are SO_3_ and CaO, with the contaminants including Cr, PO_4_^3−^, and F^−^. The loss on ignition is 20.24%. X-ray diffraction (XRD) (Figure 1) results indicate the presence of CaSO_4_·2H_2_O. The scanning electron microscopy (SEM) (ZEISS, Jena, Germany) image illustrates the plate-like structure of PG, with numerous voids present between the particles when clumped, as shown in Figure 2.

#### 2.1.2. Curing Agents

The XG is a biopolymer hydrogel of microbial origin, derived from aerobic fermentation of glucose or sucrose by Xanthomonas campestris [40]. Its monomeric chemical formula is C_35_H_49_O_29_, and its CAS Number is 11138-66-2. In the experiment, 80-mesh yellowish-white powdered xanthan gum was utilized. The SM is an organosilicon water repellent, its monomeric chemical formula is CH_3_Na_3_O_3_Si, and its CAS Number is 16589-43-8. In this experiment, we used analytical-grade liquid SM.

### 2.2. Experimental Procedure

#### 2.2.1. Preparation of Stabilized PG

The production of stabilized PG involves two different mixing methods: dry mixing and wet mixing [41,42]. For the dry mixing method, XG powder is carefully blended with dehydrated PG, followed by the addition of a predetermined amount of water for stirring. For the wet mixing method, the XG powder is dissolved in water to create a homogeneous gel, and is then mixed with PG. The gel forms quickly, as the XG is hydrophilic. However, the as-prepared gel easily encapsulates the undissolved particles, resulting in difficulty in achieving uniform consistency. Thus, the resulting composite has a high viscosity by the wet mixing method. On the other hand, the fluidity of the gel would be decreased, making it challenging to properly mix with PG [42,43]. Therefore, the dry mixing approach was chosen in this study.

The preparation of stabilized PG was followed by (i) XG and PG being thoroughly mixed together using an automatic mortar mixer; (ii) a mixture of SM and water was gradually added to the stirring mixture, and the whole mixture was continuously stirred at low-speed setting (140 rpm for the revolving action and 62 rpm for the planetary action) of the mixer for 5 min; (iii) the resulting mixture was then transferred into molds with dimensions of φ50 mm × 50 mm; (iv) the mixture was molded under static pressure using a universal testing machine; (v) the specimens were demolded and left to be cured.

#### 2.2.2. Unconfined Compressive Strength

The dependence of contents of XG, SM, and XG-SM on unconfined compressive strength of PG was evaluated. Since all percentages were weight ones, we used “%” to represent weight percentage (wt.%) to keep concise in this paper. The weight fraction was chosen to be 0.5%, 1.0%, and 1.5% for XG, SM, and XG-SM, respectively. A portion of specimens was cured at 50 °C for 5 days and was then immersed in water for 24 h. Another portion of specimens was cured at 50 °C for 6 days. The PG without XG and SM was prepared and tested for a comparison. Furthermore, the curing temperature and the curing time influence the compressive strength of the specimens [38]. In this experiment setup, a blank group (PG) was established, and stabilized PG specimens with a fixed content of SM at 1.0% and varying content of XG at 0.5%, 1.0%, and 1.5% were prepared. The curing times were 2, 4, and 6 days, and the curing temperatures were 30 °C and 50 °C. On the final day of the curing process, half of the specimens were immersed in a water tank for a period of 24 h and the other half of the specimens continued to cure in the original environment for the same duration. The unconfined compressive strength of the specimens with and without XG and SM was studied by utilizing a TC-20C type pavement material strength synthesizer (TianchangTongda, Beijing, China), with a loading rate of 1 mm/min. Furthermore, this experiment primarily focuses on the unconfined compressive strength of the specimens after immersion in water. Generally, only these data are analyzed.

In addition, standard deviation is the widely employed quantitative measure to depict the extent of dispersion within a dataset, and it serves as a crucial indicator of precision. In this experiment, the standard deviation of strength was utilized to gauge the accuracy of the strength data.

#### 2.2.3. Water Stability

Chang et al. [44] observed a pronounced decline in the strength of clay treated with biopolymers when subjected to moisture. The water stability of the roadbed greatly affects the functional capacity of pavement. In this work, the dependence of curing agent content on water stability will be studied. The water stability is evaluated by the water stability coefficient, employing Equation (1):

(1) $$\mathrm{Water}\;\mathrm{stability}\;\mathrm{coefficient}=\frac{R_{C}}{R}$$

where *R_C_* represents the unconfined compressive strength of the specimens that have been immersed in water for 24 h prior to testing. *R* signifies the unconfined compressive strength of the specimens that have been cured for the same duration in their original environment. Water stability coefficient serves as an indicator of the similarity in strength between the specimens subjected to water immersion and those that have not been immersed. A higher water stability coefficient value indicates that the strength after water immersion is closer to the strength of specimens that have not been immersed, suggesting better water stability.

#### 2.2.4. Leaching of Hazardous Substance

To investigate the release of harmful substance, an experiment on leaching was conducted following the guidelines outlined in the American testing standard method [45]. After being cured at a temperature of 50 °C for 6 days, the specimens with and without XG and SM were immersed in distilled water at a ratio of 16:1 (water to specimen mass). The solution was maintained at room temperature, and the pH was continuously monitored and adjusted to 5 ± 0.2 using a 0.5 mol·L^−1^ concentration of acetic acid. After 24 h, deionized water was added to increase the solution mass to 20 times the mass of the specimens. Finally, the solution was filtered through a 0.45 μm membrane to obtain the leaching liquid for subsequent analysis. The concentration of Cr element in the leaching liquid was determined using ICP-MS (Agilent 7700 (MS), Santa Clara, CA, USA), while the concentrations of F^−^ and PO_4_^3−^ were measured using IC (Thermo Scientific AQUION RFIC, Markham, ON, Canada).

It is important to highlight that the leaching solution obtained cannot be directly utilized for IC and ICP-MS testing. Both methods require specific pre-treatment, and the pre-treatment process varies.

Given that the sample contains water-soluble organic matter, some of this organic content dissolves in water during leaching. This poses a risk of potential damage to the instrument if directly used for IC testing. Therefore, pre-treatment is necessary. The pre-treatment process is to weigh 0.55 g of samples into a Teflon beaker, add 20 mL of 1 mol/L sodium hydroxide solution, heat on an electric heating plate for digestion for 4–5 h, and dilute to 50 mL with pure water at a constant volume. After this treatment, the concentrations of F^−^ and phosphate PO_4_^3−^ can be accurately determined. The detailed parameters of the test are as follows in Table 2.

Similarly, carbon-containing organic solutions cannot be directly employed for ICP-MS testing. These solutions must undergo digestion with nitric acid until all carbon is removed from the leaching solution. Only after this process can the Cr content be accurately measured. The detailed parameters of the test are as follows in Table 3.

#### 2.2.5. Other Characterization Methods

In order to characterize the mineral structure of the PG, phosphogypsum was carefully ground in a quartz mortar, and then the ground PG was passed through a 200-mesh sieve to collect the appropriate amount of powder for XRD testing. XRD was carried out with a Bruker D8 Advance diffractometer, with Cu Kα radiation at a scanning rate of 2°/min from 10° to 80°.

To analyze the chemical composition of PG, the powder samples prepared as described earlier for XRD can be directly employed for XRF analysis. The XRF analysis was performed using the Thermo Scientific ARL Perform ‘X Sequential X-Ray Fluorescence Spectrometer (Waltham, MA, USA). It is important to note that IC is typically used for detecting ion concentrations in liquids, and that PG is a solid material and requires specific pre-treatment for ion analysis. In this process, a measured amount of PG powder is introduced into a solvent with a fixed volume. The ion concentration in the solvent is then determined, and based on the sample mass, the ion content in the PG sample is calculated. For ion chromatography, the Thermo Scientific AQUION RFIC Ion Chromatography System (Waltham, MA, USA) was employed.

The sample, which was a mixture of 1% XG and 1% SM, was prepared and subjected to curing at 50 °C for 5 days. Subsequently, it was soaked at room temperature for 24 h. Following this, the sample was dried in a dryer at 40 °C for 48 h to ensure complete drying. The powders selected for testing were all smaller than 75 μm in size. The infrared spectra were recorded over the range of 400 to 4000 cm^−1^ using the Thermo Scientific Nicolet iS20 FTIR Spectrometer (Waltham, MA, USA).

PG samples, as well as samples mixed with 1% XG and 1% SM, and samples mixed with 1% SM alone, were prepared and subjected to curing at 50 °C for 5 days. Following the curing process, they were soaked at room temperature for 24 h. Subsequently, the cured samples were smashed into smaller blocks, each measuring less than 1 × 1 × 1 mm in size. These smaller blocks were used for the analysis of SEM. The SEM analysis was conducted utilizing a ZEISS Gemini SEM 300 (ZEISS, Jena, Germany). To enhance the quality of imaging, it was necessary to apply a thin layer of gold coating to the surface of the samples.

## 3. Results and Discussion

### 3.1. Unconfined Compressive Strength

#### 3.1.1. Effect of Curing Agent Dosage

**Figure 3:**
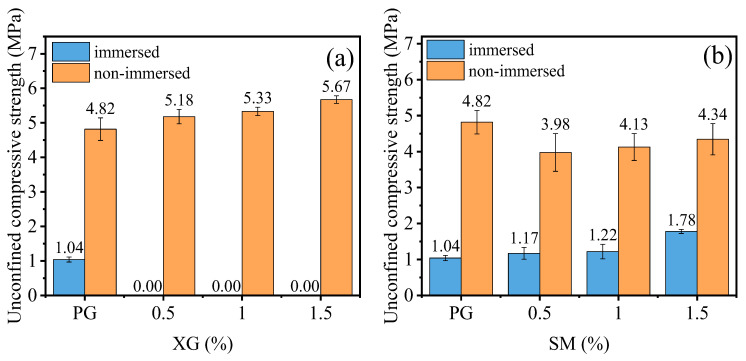
The effect of XG and SM on the unconfined compressive strength of PG: (**a**) XG; (**b**) SM.

**Figure 4:**
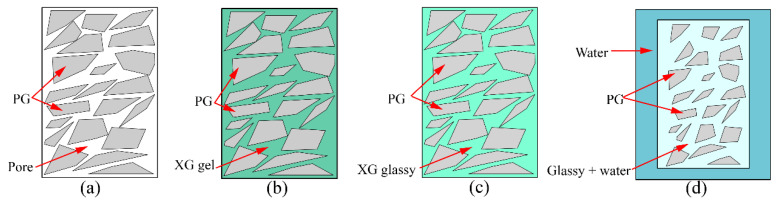
The transformation of gel and glassy: (**a**) PG with many pores; (**b**) The gel of XG fills the pores of PG; (**c**) The glassy of XG fills the pores of PG; (**d**) PG and glassy soaking in water.

**Figure 5:**
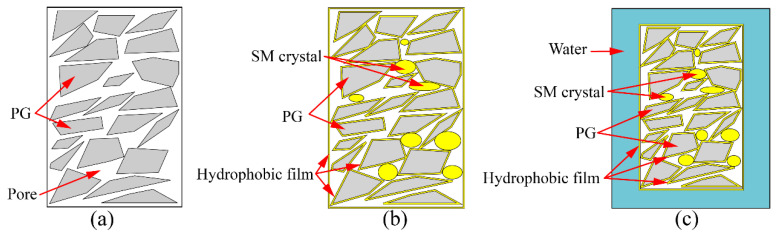
The formation of hydrophobic film: (**a**) PG with many pores; (**b**) hydrophobic film on the surface of PG particles; (**c**) PG with SM soaking in water.

The unconfined compressive strength of PG with XG and SM is shown in Figure 3a,b, respectively. Figure 3a showed that the PG without any admixture exhibits a compression strength of 1.04 MPa. However, the strength diminishes to 0 MPa with the introduction of XG. Additionally, the non-immersed specimen revealed a higher unconfined compressive strength, and the unconfined compressive strength increased with the increase in content of XG, as shown in Figure 3a. This is because the PG incorporated with XG dissolves into minute particles when immersed in water. This process occurs an hour after the immersion in water. XG, characterized as a viscoelastic and expansive biopolymer, engenders a hydrogel on the PG surface to fill the pores. Upon the dehydration of this hydrogel, XG changes into a vitreous substance, evoking a bonding effect. This vitreous material undergoes softening and swelling processes when exposed to water [46], as shown in Figure 4. Consequently, the PG with XG dissolves in water. Conversely, adding SM enhances the unconfined compressive strength of PG, as demonstrated in Figure 3b. The addition of 0.5%, 1.0%, and 1.5% increases the unconfined compressive strength by 12.5%, 18%, and 71.2%, respectively. However, the addition of SM decreases the unconfined compressive strength of non-immersed specimen, the unconfined compressive strength increases with the increase in content of SM, but it has not exceeded the unconfined compressive strength of PG. Indeed, SM creates a hydrophobic film on the surface of PG particles [47,48]. This film serves to shield PG from water erosion and dissolution, as shown in Figure 5. However, from the experimental results, the film lacks adhesive characteristics. Consequently, the incorporation of SM diminishes the unconfined compressive strength of non-immersed specimens. After immersion, the hydrophobic properties of the film contribute to decreased PG dissolution, thereby leading to an increased unconfined compressive strength.

**Figure 6:**
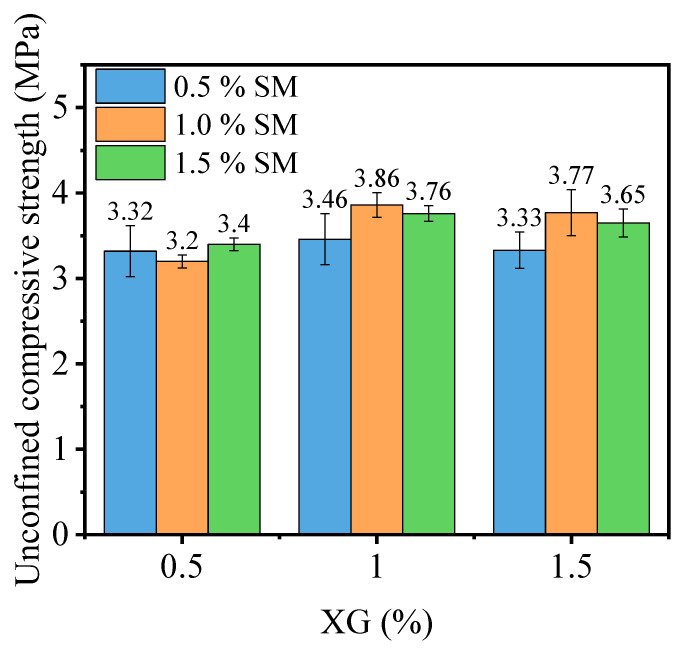
Effect of XG and SM combined on the unconfined compressive strength of PG.

Figure 6 shows the unconfined compressive strength of PG incorporated with XG and SM together. Notably, the strength of samples increases significantly when XG and SM are used together. For example, the XG dosage of 0.5%, 1.0%, and 1.5% increases the unconfined compressive strength of PG, incorporated with 1.0% SM, by 232.7%, 271.2%, and 261.5%, respectively. Increasing the dosage of XG from 0.5% to 1.5% while keeping the SM dosage at 0.5% does not have a significant impact on the strength of stabilized PG. The highest unconfined compressive strength is achieved when the dosage of SM and XG are set at 1.0%. This enhancement is due to the combination of SM and XG creating a hydrophobic film on the surface of PG and the vitreous framework formed by XG [47,48]. This hydrophobic film the PG and vitreous framework from direct contact with water, enhancing the strength of the specimens after immersion in water.

In comparison, Meskini et al. [32] utilized fly ash and lime to stabilize PG roadbed material, achieving strengths below 1 MPa at 7 days and ranging from 1–3 MPa at 30 days. In Ngo et al.’s study [33], the strength of the cement: PG = 10:90 mixture was less than 1 MPa at day 7 and remained less than 2 MPa at day 28. Shen et al.’s research [34] reported curing strengths of 2 MPa at 7 days and 8 MPa at 28 days for slag-fly ash-PG solidified material. Conversely, the minimum strength observed for stabilized PG mixed with XG and SM after curing at 50 °C for 6 days and subsequent immersion in water was 3.33 MPa. This surpasses the strength of traditionally stabilized inorganic PG materials at the 7-day mark. When considering strength as the primary criterion, stabilized PG demonstrates greater potential than the conventional use of cement, lime, and fly ash.

#### 3.1.2. Mechanism of Unconfined Compressive Strength Increasing

**Figure 7:**
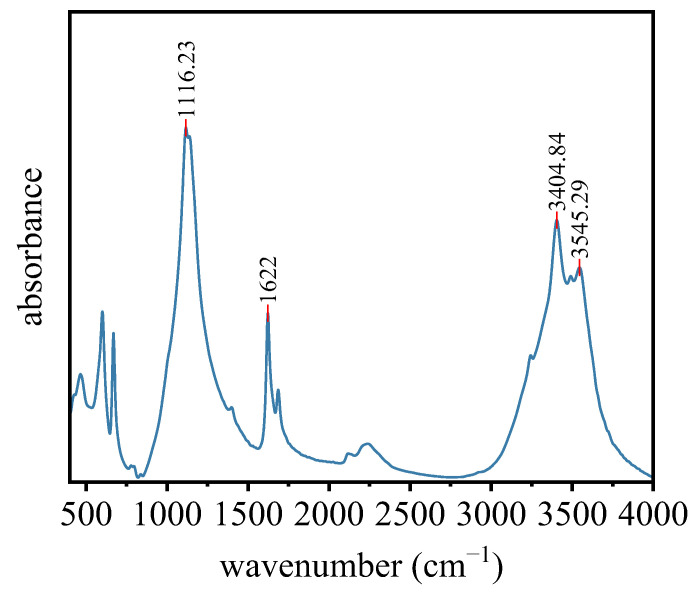
Fourier Transform Infrared Spectrum of PG with 1.0% XG and 1.0% SM.

According to Figure 6, the optimal concentration of both XG and SM is 1.0% regarding the unconfined compressive strength. Thus, this section aims to reveal the mechanism behind this enhancement by microstructural and compositions analysis. The Fourier Transform Infrared Spectrometer (FT-IR) of the PG with 1.0% XG and 1.0% SM is shown in Figure 7. The absorption peak at 1116.23 cm^−1^ and 466.12 cm^−1^ corresponds to the hydrophobic membrane Si-O-Si bonds [49,50]. The absorption peaks at 3545.29 cm^−1^ and 3404.84 cm^−1^ were stretching vibrations of -OH [47]. The peak at 1622 cm^−1^ is attributed to the characteristic absorption of the dissociated carboxyl group -COO- [51]. These results confirm the presence of XG and SM in the composites.

**Figure 8:**
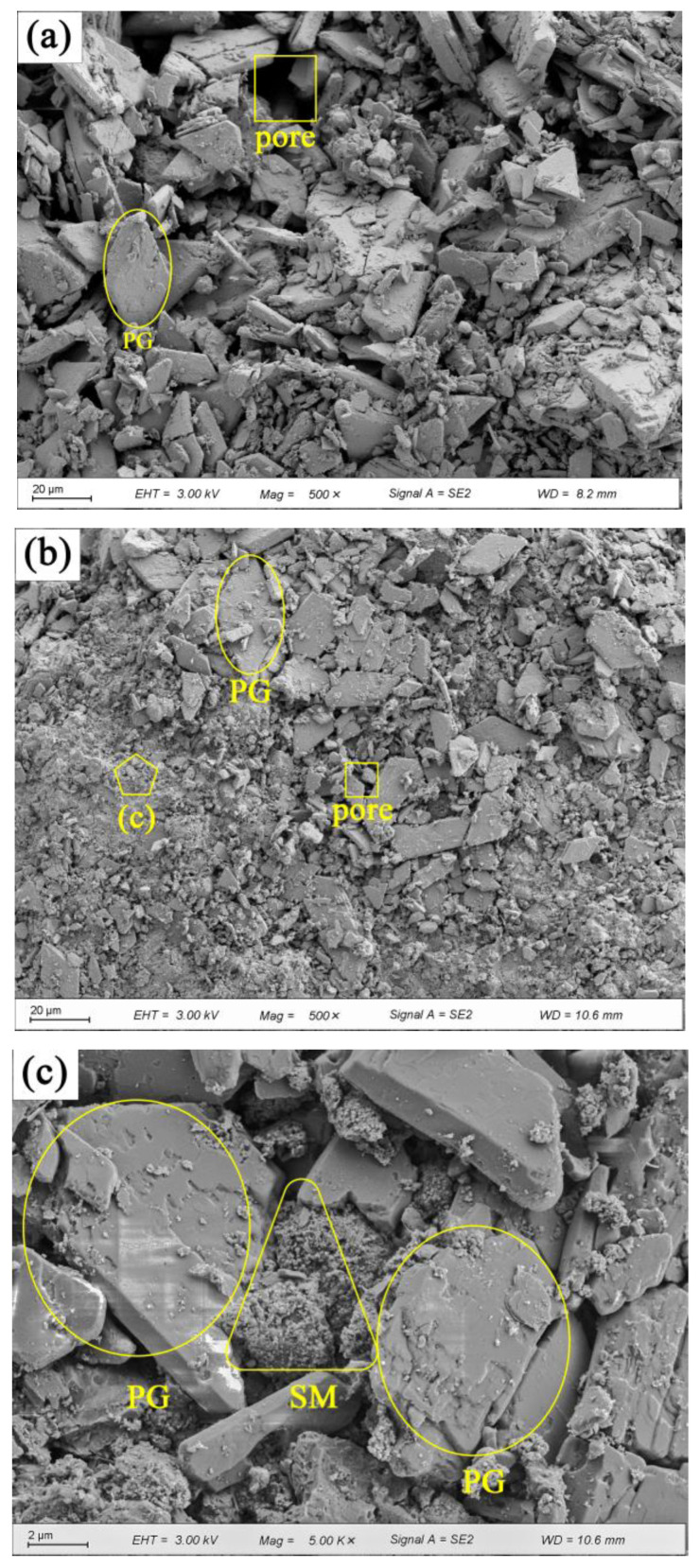
Morphology of PG with and without SM: (**a**) PG without SM, 500× (20 μm); (**b**) PG with 1.0% SM, 500× (20 μm); (**c**) PG with 1.0% SM, 5000× (2 μm).

Figure 8 displays the SEM images of PG with and without 1.0% SM. Figure 8a shows that the flake-like shaped PG particles are randomly distributed. The presence of numerous voids and weak bonding (as shown by the loose packing of PG particles) allows water to easily infiltrate the interior. Consequently, the CaSO_4_·2H_2_O dissolves, leading to a reduction or even elimination of cohesion [31]. This ultimately results in a strength of only 1.04 MPa for the PG without any admixture after being soaked in water, as shown by Figure 3a. Figure 8b presents the SEM image of PG with 1.0% SM. In comparison to Figure 8a the number of voids between the PG particles appears to be reduced, and visible SM crystals are evident (Figure 8c). Figure 8b,c show that SM goes into voids and then crystallize, causing slight expansion within the voids. Additional, SM creates a hydrophobic film on the surface of PG particles [47,48], as shown in Figure 5, but this contributes little to the strength, the increase in unconfined compressive strength after immersion is due to the reduction in porosity caused by crystallization and the hydrophobic effect of SM, the main reliance is on the intrinsic cohesive strength of the PG itself, this is demonstrated in Figure 3b, where the strength of the immersed sample is similar to that of PG after the addition of SM.

**Figure 9:**
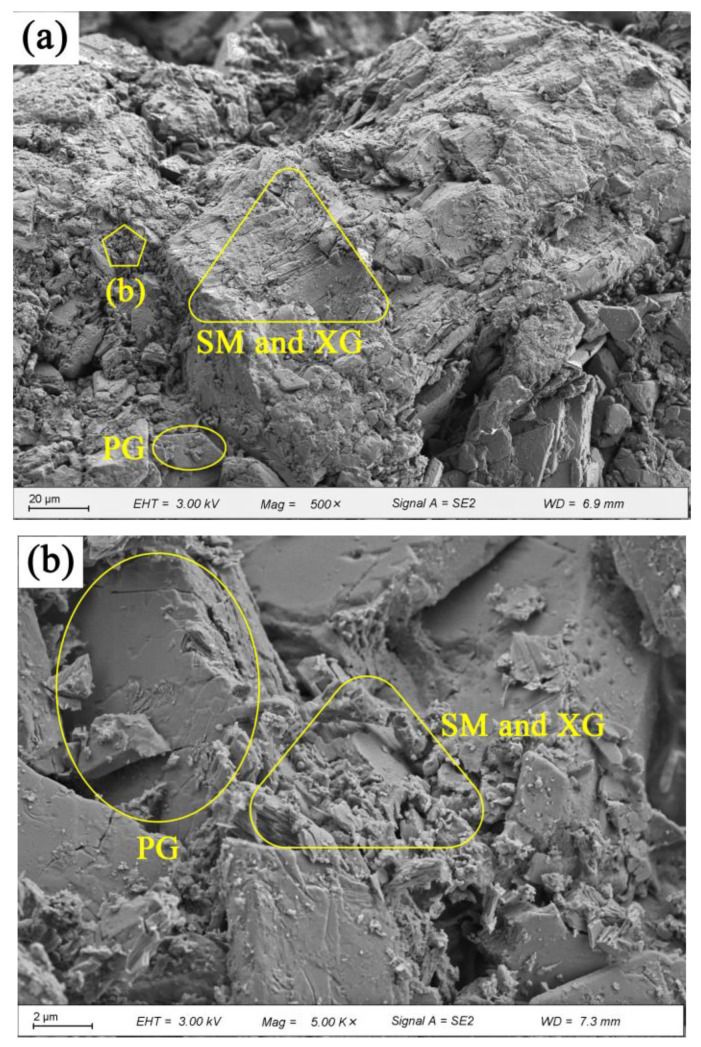
Morphology of PG incorporated with 1.0% XG and 1.0% SM: (**a**) 500× (20 μm); (**b**) 5000× (2 μm).

SEM images of PG with 1.0% XG and 1.0% SM are shown in Figure 9. The images reveal that the voids are rarely observed. XG main function to bind the PG particles together while simultaneously filling the gaps between them, thereby decreasing porosity and enhancing strength. The XG hydrogel forms a protective layer on the surface of PG particles, which transforms into a glassy state after dehydration [52,53]. Similarly, SM generates a small quantity of crystals and a hydrophobic film, which prevents PG and XG from directly contacting water [47,54]. Moreover, the glassy substances and crystals fill the voids, leading to the formation of a cross-linked network structure and an increase in the strength of stabilized PG [41,55]. However, the excessive addition of XG, exceeding 1.0%, causes the immersed stabilized PG to swell and reduces its compactness due to the expansive nature of XG, resulting in a slight decrease in strength.

**Figure 10:**
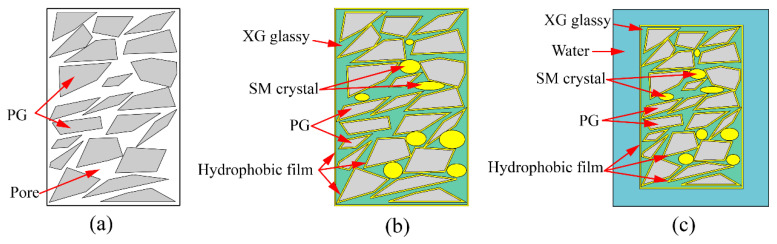
Mechanism of unconfined compressive strength increasing: (**a**) PG with many pores; (**b**) PG incorporated with XG and SM; (**c**) PG incorporated with XG and SM soaking in water.

The mechanism diagram illustrating the strengthening of unconfined compressive strength is depicted in Figure 10. In Figure 10a, it is evident that pores exist within the compacted PG, which aligns with the observations made in Figure 8a. These pores are a contributing factor to the lower unconfined compressive strength (only 1.04 MPa) observed in PG. Subsequently, with the introduction of both XG and SM into PG and subsequent curing, significant transformations occur. The glassy substances formed as a result of the dehydration of XG effectively fill these pores and establish bonds between PG particles (Figure 9b). Concurrently, crystals generated by SM partially occupy these pores (Figure 8c), while SM creates a hydrophobic film on the surfaces of both XG and PG, as shown in Figure 10b. Ultimately, when the sample is immersed in water, the hydrophobic film created by SM acts as a barrier, preventing water from infiltrating the sample’s interior. This prevents the glassy substance formed by XG from softening and transitioning into a gel-like state. Consequently, even after 24 h of immersion in water, the sample retains a certain level of strength (Figure 6), as shown in Figure 10c. However, it is important to note that excessive XG can lead to pore support, which reduces density and consequently results in decreased strength. This phenomenon is reflected in Figure 6.

#### 3.1.3. Effect of Temperature and Curing Time

**Figure 11:**
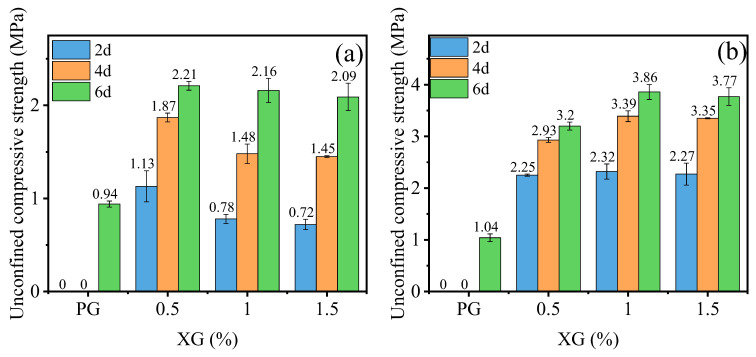
The influence of temperature and curing time on unconfined compressive strength of stabilized PG with different XG dosage: (**a**) 30 °C; (**b**) 50 °C.

Figure 11 shows the results of the effects of curing temperature and time on the unconfined compressive strength of PG incorporated with various dosages of XG. It can be observed that at both temperatures, the unconfined compressive strength of PG without XG after 6 days of curing is 0.94 MPa and 1.04 MPa for 30 °C and 50 °C, respectively. According to the literature [31], strength is attributed to the existing of inherent cohesion and additional cohesion caused by capillary effect when the water content is low, and the moisture content of PG remains relatively high after 2 and 4 days of curing. At this point there is only a relatively low strength, after immersion in water, as the strength is completely lost. Additionally, at each content of XG, the unconfined compressive strength increases with increasing curing time and curing temperature. Specifically, at temperature and curing time of 30 °C and 6 days, the unconfined compressive strength of PG with XG concentrations of 0.5%, 1.0%, and 1.5% is increased by 95.6%, 177%, and 190.3%, respectively, which is higher than those at temperature and curing time of 30 °C and 2 days. Similarly, for 50 °C and 6 days, the unconfined compressive strength of PG with XG concentrations of 0.5%, 1.0%, and 1.5% is increased by 42.2%, 66.4%, and 66.1%, respectively, which is higher than those for 30 °C and 2 days. Furthermore, for all XG concentrations and curing time, the unconfined compressive strength of sample at 50 °C is higher than that at 30 °C, with an average increase of 114.7%. Finally, when the curing time and temperature are the same, the unconfined compressive strength of PG with 1.5% XG is 7.7% slightly lower than that with 1.0% XG. These results indicate that proper addition of XG, longer curing time, and higher curing temperature contribute to unconfined compressive strength of stabilized PG. Additionally, an optimal XG concentration, such as 1.0%, can yield the highest unconfined compressive strength.

The conversion of XG gel on the surface of PG particles into a glassy state is a time- and temperature-dependent dehydration process [38,52]. When the curing time is short, a significant portion of the XG gel remains in its gel phase. The gel phase provides limited bonding effect, leading to a lower macro-level unconfined compressive strength. With increasing curing time, more and more gels transform into glassy state, consequently enhancing the unconfined compressive strength. The transformation process is accelerated by higher temperatures as the thermal energy serves as the activation energy of this process. This is consistent with the statement above—that the unconfined compressive strength of samples cured at 50 °C is higher than that cured at 30 °C at the same XG concentration and curing time. Excessive XG content leads to the expansion of stabilized PG, resulting in reduced compactness. Consequently, PG with 1.5% XG exhibits slightly lower unconfined compressive strength compared to that of 1.0% XG.

### 3.2. Water Stability

#### 3.2.1. Effect of Curing Agent Dosage

**Figure 12:**
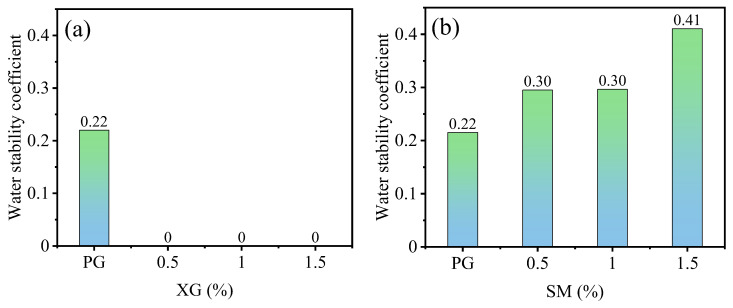
The effect XG and SM on the water stability coefficient of stabilized PG: (**a**) XG; (**b**) SM.

The effect of XG and SM content on the water stability coefficient of stabilized PG is illustrated in Figure 12. The water stability coefficient of PG without any admixture is 0.22, as shown in Figure 12a,b. However, upon the addition of XG, the water stability coefficient reaches 0 across all concentrations. On the other hand, Figure 12b indicates that the water stability coefficient increases with the rise of SM content. Specifically, at SM concentrations of 0.5%, 1.0%, and 1.5%, the water stability coefficient demonstrates a respective increase of 37.1%, 37.7%, and 90.8% compared to the PG without any admixture.

The effect of XG on the water stability is consistent to that on the unconfined compressive strength. After being submerged in water, PG samples experience a decrease in cohesion and a significant loss of strength compared to dry samples, as indicated by the low water stability coefficient 0.22. However, when XG is added to the samples, the dehydrated XG forms a gel-like substance that swells in the presence of water. This swelling causes the samples to dissolve and disperse completely, resulting in no water stability coefficient (Figure 12a) and unconfined compressive strength (Figure 3a). This phenomenon is similar to previously reported results—that treating soil with polymers leads to a notable decrease in strength in humid environments [44,56].

When SM makes contact with water and CO_2_, SM breaks down to form methyl silicic acid, which quickly polymerizes to create polymethylsiloxane. The methyl groups in polymethylsiloxane are of low affinity to water molecules, as the polymethylsiloxane coating generated on the surface provides hydrophobicity and consequently protects the substrate from water infiltration [47,54]. This film effectively prevents water from penetrating into the interior of OPCA, thereby significantly improving the water stability of stabilized PG. As shown in Figure 12b, the water stability coefficient at 1.5% SM content is 86% higher than that without SM.

**Figure 13:**
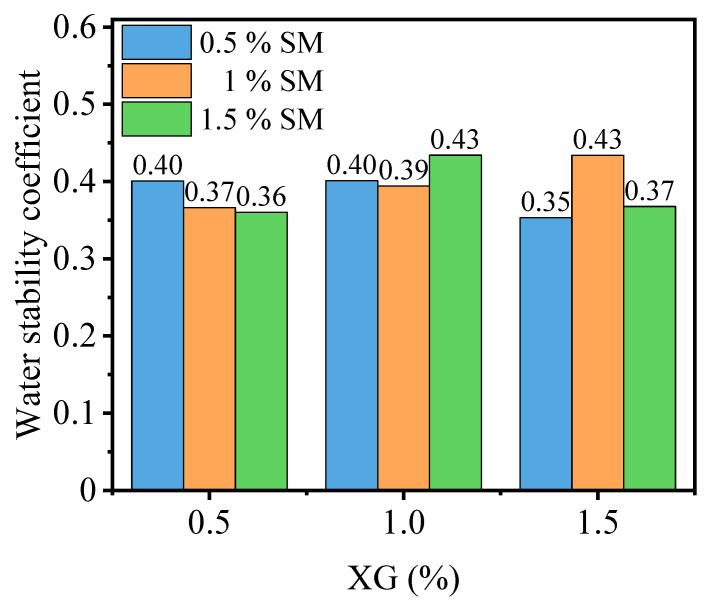
Effect of XG and SM combined on the water stability.

The results presented in Figure 13 demonstrate that the incorporation of both XG and SM into PG leads to a significant improvement in water stability. The specific effects depend on the concentrations of XG and SM used. For PG with 0.5% SM content, the water stability coefficient initially remains stable and then decreases as the XG content increases. This suggests that the addition of XG decreases the water stability of stabilized PG when combined with a low concentration of SM. The relative enhancements compared to the PG are 82.1%, 82.3%, and 60.5% for XG contents of 0.5%, 1.0%, and 1.5%, respectively. On the other hand, for PG with 1.0% SM content, the water stability coefficient increases as the XG content increases. This indicates that the addition of XG further improves the water stability of PG when combined with a higher concentration of SM. The relative enhancements compared to the PG are 66.4%, 79.1%, and 97.2% for XG contents of 0.5%, 1.0%, and 1.5%, respectively. Similarly, for PG with 1.5% SM content, the water stability coefficient initially increases and then decreases as the XG content increases. This suggests that the combination of XG and a higher concentration of SM can enhance the water stability of stabilized PG to a certain extent, but excessive XG content might have a negative effect. The relative enhancements compared to the PG are 63.8%, 97.3%, and 67.1% for XG contents of 0.5%, 1.0%, and 1.5%, respectively. Overall, the co-blending of XG and SM in stabilized PG shows promising results in terms of improving its water stability, with the specific effects depending on the concentrations of both additives.

During co-blending, the glassy substance formed by XG and the crystalline structure of SM contribute to reducing the porosity and limiting the interconnected voids, thus impeding the ingress of water into the interior of stabilized PG. Furthermore, the hydrophobic polymethylsiloxane coating formed by SM acts as a protective coating, encapsulating both the glassy substance formed by XG and the PG particles, thereby enhancing water stability [47,54]. This can be concluded by comparing Figure 12a and Figure 13 when, upon the addition of XG, the water stability coefficient reaches 0 across all concentrations. When SM is added into the mixture of PG and XG, the water stability coefficient can exceed 0.35.

It is important to note that there exists an optimum blending ratio of XG and SM. When the polysiloxane film formed by SM fails to completely encapsulate the glassy substance and PG, a portion of the glassy substance softens upon water immersion, and partial dissolution of PG occurs, resulting in a diminished water stability coefficient. Conversely, when the polysiloxane film formed by SM fully encapsulates the glassy substance and PG, the glassy substance remains unaffected by water softening, thus yielding a favorable water stability coefficient. However, excessive SM content weakens the bonding between glassy substances and between glassy substances and PG, leading to a decrease in water stability coefficient.

#### 3.2.2. Effect of Temperature and Curing Time

**Figure 14:**
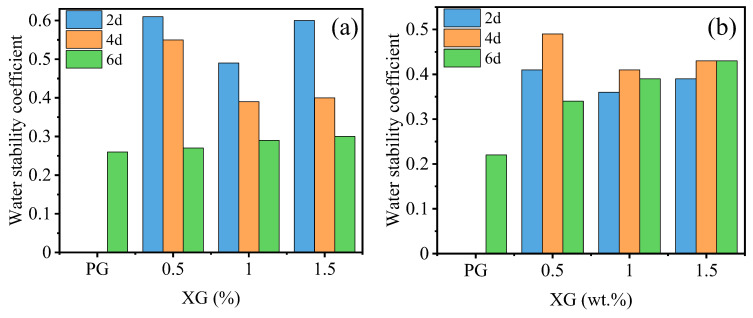
Influence of temperature and curing time on water stability coefficient of PG with different XG dosage: (**a**) 30 °C; (**b**) 50 °C.

Figure 14 illustrates the effect of temperature and curing time on the water stability coefficient of stabilized PG. It can be noted that the PG cured for 2 and 4 days at both temperatures exhibited a water stability coefficient of 0, indicating no water stability. However, when cured for 6 days, the PG displayed a relatively low water stability of 0.26 and 0.22 at both temperatures, respectively. Furthermore, at a curing temperature of 30 °C, the water stability coefficient of stabilized PG samples with the same XG content decreases with the curing time. Compared to the specimens cured for 2 days, the water stability coefficient of stabilized PG samples with 0.5%, 1.0%, and 1.5% XG content is 55.7%, 40.8%, and 50% lower than those cured for 6 days, respectively. In addition, at a curing temperature of 50 °C, the water stability coefficient of stabilized PG samples cured for 4 and 6 days with the same XG content shows little difference, both exhibiting an increase compared to the samples cured for 2 days. However, the water stability coefficient is lower for samples cured for 6 days than those cured for 4 days. Compared to the samples cured for 2 days, the water stability coefficient of stabilized PG samples cured for 4 days is increased by 19.5%, 13.9%, and 10.3% with XG content of 0.5%, 1.0%, and 1.5%, respectively. At both temperatures, the water stability coefficient of stabilized PG samples cured for 2 and 4 days initially decreases and then increases with increasing XG content, while the coefficient of which cured for 6 days increases with increasing XG content. Overall, these results indicate that both temperature and curing time have significant effects on the water stability of stabilized PG, and the incorporation of XG can influence the water stability coefficient in different ways depending on the curing conditions.

It is important to highlight that in Figure 14a,b, a discernible pattern emerges where the water stability coefficient of specimens subjected to a 6 day curing period exhibits a direct correlation with increasing XG content. Our discussion will now be centered around these two graphical representations. Notably, this trend becomes particularly prominent when the curing takes place at a temperature of 50 degrees Celsius (Figure 14b). For PG, the water stability coefficient stands at 0.22. When the XG content is set at 0.5%, 1.0%, and 1.5%, the corresponding water stability coefficient escalates by 54.55%, 77.27%, and 95.45%, respectively, in comparison to PG. This marked upward trend underscores a significant enhancement. However, specimens cured for 2 days and 4 days did not exhibit a clear trend in water stability coefficient with increasing XG content. This is attributed to the intricate interplay of numerous variables, such as temperature, specimen properties, and content, impacting the conversion rate between the gel and glassy states of XG within this experimental context. During the 2vday and 4-day curing periods, the gel and glassy state components contend with each other. However, when the curing duration extends to 6 days, the gel state has largely transformed into glassy state components. As a result, the absence of a pronounced trend during the 2-day and 4-day curing periods was observed.

Moreover, upon juxtaposing the water stability coefficient after a 6 day curing time at both 30 °C and 50 °C, a noteworthy result emerges. The exception of PG cured at 30 °C, which exhibits a lower water stability coefficient than its counterpart cured at 50 °C, and the water stability coefficient of stabilized PG specimens at 50 °C surpassed those cured at 30 °C, spanning across different levels of XG content. This observation indirectly underscores that elevating the curing temperature contributes to an augmentation in the water stability of stabilized PG.

Due to the pronounced increasing trend of the water stability coefficient of stabilized PG after curing for 6 days with increasing XG content, an attempt was made to discuss the XG content when the water stability coefficient approaches 1 through data fitting. Based on the data in Figure 14, the fitting equation for stabilized PG cured at 30 °C for 6 days can be expressed as Equation (2):

(2) y=0.028x+0.259

The fitting equation for stabilized PG cured at 50 °C for 6 days can be expressed as Equation (3):

(3) $$y=\;-0.08x^{2}+0.256x+0.233$$

**Figure 15:**
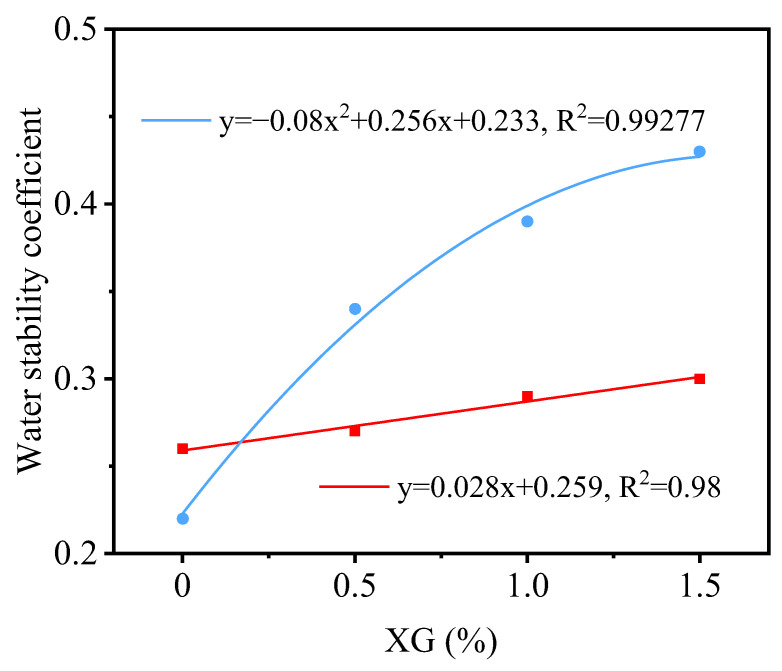
Function fitting comparison.

In the equations, x represents the XG content and y represents the water stability coefficient. Validation using experimental data is shown in Figure 15, where Equations (2) and (3) exhibit good fits to the water stability coefficient of stabilized PG cured for 6 days at 30 °C and 50 °C, respectively.

Primarily, as depicted in Figure 11a, the unconfined compressive strength exhibits a decrease with increasing XG content post-incorporation. Excessive XG introduction can adversely impact compactness, culminating in compromised strength. Secondarily, as discussed earlier, an optimal ratio exists for the concurrent application of XG and SM. When considering cost-effectiveness, the utilization of a substantial XG quantity, coupled with the requisite SM amount, might not be financially feasible. Lastly, as highlighted earlier, the curing process of XG necessitates specific time and temperature conditions. Should temperature remain constant, higher XG content would mandate extended curing periods, potentially leading to project delays.

It is important to note that in the ongoing experiment, stabilized PG featuring 1.5% XG content attains a water stability coefficient of 0.43 following a 6-day curing process at 50 °C. Considering the insights provided by Figure 11b and factoring in economic considerations, it is advisable to opt for the inclusion of 1.5% XG for practical implementations. This XG content strikes a judicious equilibrium between the enhancement of water stability and the pragmatism of cost-effectiveness.

### 3.3. Leaching of Hazardous Substance

The results of the analysis of hazardous elements in the leachate of the PG and stabilized PG specimens are shown in Table 4. It is evident that XG and SM (fixed dosage of 1.0%) effectively immobilize the hazardous elements, leading to a reduction in their leaching. However, the leaching behavior is not directly proportional to the XG concentration. Comparing to the PG, when the concentration is 1.0% for SM and 0.5%, 1.0%, and 1.5% for XG, the leaching of Cr in stabilized PG is reduced by 74.4%, 75.2%, and 74.7%, respectively. Similarly, the leaching of F^−^ is reduced by 84.0%, 82.1%, and 82.4%, while the leaching of PO_4_^3−^ is reduced by 58.11%, 65.04%, and 61.22%, respectively.

The combination of XG and SM effectively reduces the leaching of heavy metals and hazardous ions in PG due to two main factors. First, the interaction between XG and water forms a viscous water gel, which leads to physical adsorption and chemical binding of the hazardous substances [57,58]. Second, as evident from the preceding text, the glassy material formed during the dehydration of XG fill the voids, while the glassy film from XG and the hydrophobic film from MS on the particle surface hinder the migration of hazardous substances, further reducing leaching. Additionally, 0.5% XG can completely adsorb and immobilize harmful substances in PG, so there is no significant correlation observed between the leaching and the concentration of XG.

In other research, Wu et al. [59] utilized either calcium carbide slag or lime as alkali neutralizers for PG, along with polyferric sulfate or polyaluminum chloride as oriented stabilizers. Their experimental findings demonstrated that the leaching concentrations of phosphorus (P), fluorine (F), and heavy metals from PG were all below 0.5 mg/L, 10 mg/L, and 0.1 mg/L, respectively. In a separate study by Ngo et al. [33], they examined the leaching of pollutants from a sample with a cement: PG ratio of 10:90. The results indicated that the concentration of total phosphorus in the leaching solution was 2.3 mg/L, and the concentration of fluoride was 13.2 mg/L. The experimental data presented in Table 4 show that the leaching concentrations of heavy metals, fluoride (F), and phosphate (PO_4_^3−^) in stabilized PG are all below 0.462 μg/L, 2.821 mg/L, and 9.939 mg/L, respectively. It is important to note that the effectiveness in reducing the leaching of harmful substances is influenced by the origin and composition of the PG, which can vary significantly. If the leaching levels of harmful substances meet local regulatory standards, it is considered suitable for practical applications.

## 4. Conclusions

The study explored the use of XG and SM as curing agents for PG and investigated the resulting properties of the stabilized PG. Key parameters such as unconfined compressive strength, water stability coefficient, and leaching behavior were evaluated. The mechanisms responsible for the strength development of stabilized PG were further examined using FT-IR and SEM. The conclusions are drawn as follows:

(1) Incorporating XG and SM together in PG yields positive effects on its unconfined compressive strength, water stability coefficient, and the reduction of hazardous substance leaching. Notably, the addition of SM improves strength and water stability, whereas the addition of XG decreases the strength and weakens water stability. The most favorable outcomes can be obtained when the concentration is 1.0% for XG and SM individually. The presence of a glassy material formed by XG fills the voids in PG and bonds the particles together, while the hydrophobic film formed by SM protects the PG and XG from water erosion, leading to improved water stability and strength of stabilized PG.
(2) The strength of stabilized PG increases with curing time curing temperature, while the water stability coefficient exhibited varying trends depending on the curing temperature. The curing temperature and time significantly influence the rate of XG gel transforming into a glassy state, with faster transformation being more favorable.
(3) The dual incorporation of XG and SM also remarkably reduces the leaching of hazardous substances from PG, particularly at both concentrations of 1.0%. This reduction is achieved through physical adsorption, chemical binding, and physical blocking of the release of hazardous substances by XG and SM.
(4) The results verify the feasibility of XG and SM stabilized phosphogypsum roadbed materials, which are more environmentally friendly than traditional inorganic cementing materials, but there are also some defects. Future research can focus on improving the curing process, evaluating long-term performance, and assessing environmental impacts. Addressing these aspects will enable the utilization of XG and SM as curing agents for PG-based materials to offer environmentally friendly and economically feasible solutions for sustainable infrastructure development.

## Figures and Tables

**Table 1:** Chemical composition of PG.

Composition	SO_3_	CaO	SiO_2_	Al_2_O_3_	P_2_O_5_	Fe_2_O_3_	F^−^	Cl^−^	Cr_2_O_3_
Component (wt.%)	43.47	28.79	4.72	0.877	0.615	0.358	0. 14	0.0060	0.0026

**Table 2:** The operating parameters of determination of elements by IC.

Method Parameters	
eluent type	KOH
eluent flow (L/min)	1.0
column	AS19
Temperature (°C)	30
suppressor	AERS-4 mm
Current (mA)	50
detector	Conductivity detector
injection volume	25 μL

**Table 3:** The operating parameters of determination of elements by ICP-MS.

Method Parameters	
Plasma gas flow (L/min)	0.5
Nebulizer and auxiliary gas flow (L/min)	1.50
RF Power (kW)	1.55
Integration time (s)	0.9
Replicates per sample	3
Mode of operation	He

**Table 4:** The level of harmful substance dissolution in PG and stabilized PG samples.

	Cr/(μg·L^−1^)	F^−^/(mg·L^−1^)	PO_4_^3−^/(mg·L^−1^)
PG	1.807	15.784	23.731
1.0% XG	0.462	2.533	9.939
2.0% XG	0.449	2.821	8.297
3.0% XG	0.458	2.780	9.202

## Data Availability

Not applicable.
